# Behavioral change communication strategy vital in malaria prevention interventions in rural communities: Nakasongola district, Uganda

**Published:** 2012-12-25

**Authors:** Margaret Mugisa, Abel Muzoora

**Affiliations:** 1 African Medical and Research Foundation (AMREF), Uganda Country Office

**Keywords:** Behavioral Change Communication, malaria, Uganda, knowledge, prevention, intervention

## Abstract

**Introduction:**

Malaria is a leading killer disease in Uganda and it accounts for significant morbidity in pregnant women and children. Pregnant women are more susceptible to malaria, which causes adverse effects including abortion, low birth weight and maternal anaemia. Children with severe malaria frequently develop one of these symptoms including: severe anaemia, respiratory distress, Prostration, convulsions and cerebral malaria. Due to the severity of the disease there is need for multiple interventions to reduce the disease burden. African Medical and Research Foundation (AMREF) adopted community based approaches to improve malaria prevention. Behavioral change communication (BCC) was fundamental at every process of Project implementation. This paper shares AMREF's experience in using BCC strategies amidst other interventions in malaria prevention approaches involving use of insecticide treated nets and environment management.

**Methods:**

AMREF through a Malaria project (2007-2010) in Nakasongola district supported BCC activities through training, community mobilization, mass media, health promotion and advocacy. Program performance was measured through baseline and evaluation surveys in 2007 and 2010.

**Results:**

The final project evaluation indicated improvement from baseline values as follows: knowledge on prevention of malaria among school children from 76.6% to 90%, under five children sleeping under bed net the previous night from 51% to 74.7%, and from 24% to 78% among pregnant women.

**Conclusion:**

Mobilization of malaria prevention interventions can be successful once BCC approaches are adequately planned and coordinated. Malaria prevention through BCC strategies are likely to be more effective with integration of other malaria interventions, and involvement of community based structures.

## Introduction

Nakasongola district is a part of Luwero triangle in the central part of Uganda on Bombo-Gulu Road 114 Km North of Kampala. It boarders Apac District in the North, Mukono in the East, Masindi District in the West and Luwero District in the South. It is covered by an area of 3424sq.km of which 321.6 sq. Km is occupied by swamps and a lake. The District total population according to the 2002 population and housing census is 127,064, of which 63,799 were female and 63,265 male. The district has 15 Health Centre IIs and 07 Health Centre IIIs [1].

The district has some of the worst health status indices in the country; fertility rate is 6.8%, immunization coverage of 43% [2] and Malaria accounts for 46% of the disease burden in Nakasongola and 26% of the total burden in Uganda [3]. Maternal mortality rate stands at 505/100,000 and under 5 mortality rate is at 129/1000 live births [4]. Nakasongola District lies in a malaria endemic area with year-round high malaria transmission and relatively little seasonal variation. The district's strategy for the prevention and control of malaria includes use of Insecticide Treated Nets, Home Based Management of Fever and social mobilization, the district has not been able to fully implement the strategy due to limited resources.

AMREF in partnership with the District local government initiated a project of Enhancing the Capacity for Prevention and Treatment of Malaria from 2007-2010. This was a response to strengthen community support structures scale up malaria prevention and treatment interventions in Nakitoma and Kakooge Sub counties in Nakasongola district. The Project aimed at addressing insufficient community efforts to address malaria control and prevention, limited ITN distribution outlets at community level; inadequate community malaria treatment seeking behaviour/low levels of awareness among the population and weak coordination, collaboration and advocacy among civil society organizations involved in malaria and other healthcare interventions.

## Methods

**Implementation strategies:** Behavioral Communication Change (BCC) strengthens all strategic components of malaria control and prevention programmes by supporting delivery of interventions like case management improvement, Integrated Vector Management and programme management [5]. BCC is the deliberate use of communication to promote positive health outcomes, based on proven theories and models of behavior change [6]. The strategy is an interactive process with communities that develops tailored messages to increase knowledge of malaria for instance, stimulating social and communication dialogue, promoting essential attitude change and creating demand for information and services [6]. Its primary goal is to facilitate positive behavior change and capacity building through the provision of correct and relevant information to empower people for more effective decision-making in utilization of health services [7].

Behavioral Communication Change is at the centre of all the community based approaches fronted by AMREF at each implementation process in the project. The interventions used in the project included, baseline and end line surveys, capacity building; community system strengthening and community partnering. These strategies were vital in the implementation process of malaria prevention methods in the community.

### Baseline and end-line surveys

**Baseline:** On the commencement of the project in 2007 a baseline survey was carried out by an external consultant to provide situation assessment information to support project indicator development and planning. At the time of writing this paper, detailed information on methodology of baseline survey could not be accessed.

**End line:** In 2010 end line survey was conducted after a period of three years. The end line targeted various stakeholders to provide information critical for the Project. The Project team, Parish Malaria Control Groups, Persons Living with Aids, the in charges of Health Centres II and III, the District Health Team, District Malaria Coordination Committee (DMCC), District Malaria Focal Person, heads of NGOS and CBOs were purposively sampled for the study because they were considered as key informants. They were also interviewed in order to obtain as much information from these key informants as possible. The end line employed a cross-sectional survey design to select respondents from the two sub-counties (Nakitoma and Kakooge) survey and one sub-county (Wabinyonyi) was used as a control area in order to derive the true impact of the Project activities [8]. The design was considered appropriate because the evaluation sought a cross-section of views of various stakeholders from various parishes regarding project implementation. Both qualitative and quantitative data was sought in order to improve validity of the findings. The study was conducted in two parishes from each of the sub-counties of Kakooge in Nakasongola County, Nakitoma in Budyebo County and Wabinyonyi in Buruuli County. All the six parishes selected have reliable government Health Centre records. The parishes in Kakooge and Nakitoma were selected because it is where AMREF implemented the malaria project while the two parishes in Wabinyonyi acted as the control group during the evaluation [9].

In the sub-counties of Kakooge and Nakitoma where the Project was implemented, 16 villages were stratified and randomly chosen on the basis of criteria such as presence of a health centre and distance from the sub-county headquarters i.e. eight villages per parish. At least two of the villages were chosen from distant places. This gave a total of 16 out of 79 villages (20.3%) where the Project is operating, and this is representative according to Krejcie and Morgan table for determining sample size. In Wabinyonyi sub-county, two villages in each of the two parishes were sampled for the study [10].

In each village, views of mothers of U5 children, pregnant mothers, school going children and people having HIV/AIDS and the associated stakeholders such as community members and their leaders, health workers, school teachers, Community Health Teams were sought. The evaluator employed a variety of approaches so as to triangulate the information obtained in order to increase its validity. The study relied on primary and secondary sources of data [11].

### Capacity-Building as community empowerment strategy

The project built the capacity of the local leaders, health workers, community health workers and Community-based Organizations. The purpose was to improve their knowledge and skills to prioritize, plan and manage malaria interventions.

**Health workers:** AMREF worked with health workers comprising of senior nursing officers, midwives, registered nurses, clinicians and laboratory technologists as technical personnel supporting and implementing health interventions in the community. All the earlier mentioned health cadres were trained using Ministry of health (MOH) curriculum in areas of case management, diagnosis of complicated, uncomplicated malaria and the use of rapid diagnosis treatment.

**Community health workers:** Community health worker is a government strategy to strengthen the delivery of health services [12]. They are groups of people who link communities with the formal health system and are meant to offer primary health care [13] at community level. They advocate for increased community uptake of prevention interventions such as immunization, essential nutrition and use of Insecticide-treated Nets [13]. AMREF trained CHWs using approved MoH training manual. They were also mentored on job as they implemented the project's health interventions. The community health workers were trained in managing complicated and uncomplicated malaria. They played a big role in referring children under 5years to health facilities for prompt treatment. They carried out home visits regularly in their respective villages. CHWs used Interpersonal communication a component of BCC (health education) in which information is provided to communities in person and their health questions clarified on spot [14].

**Community based Organizations (CBOs):** The project mentored the local partners and CBOs to engage in evidence-based advocacy activities relating to malaria prevention and treatment. The CBOs were trained in proposal development, financial management, monitoring and community mobilization and malaria prevention interventions. The CBOs were instrumental in mobilizing for health interventions using music, dance and drama, open air discussions and staging film shows in the community. Folk media has its roots in the culture and tradition of the communities. This was done to provide education and entertainment [15].

**Parish Malaria Coordination Groups (PMCGs):** These were groups of persons selected from the CHWs; who were responsible for monitoring CHWs as well as collecting data and sharing it with the project team [16]. They were equipped with knowledge on monitoring of malaria interventions. This group held regular meetings with CHWs to share information and updates from the health facility for instance utilization of data and availability of drugs at the health facility. They also supported the CHWs drama groups which sensitized communities through edutainment on malaria prevention strategies. They shared their findings during sub county review meetings at Sub county level.

### Community participation

Participation can be understood as a process that contributes to reinforcing community power [17]. Partner communities’ participation in an intervention greatly increases the chance of success. Community participation should not only focus on a make for people or population approach but rather make with them. This participation negotiation approach should be assumed at all levels. AMREF used a Community Based Health Care (CBHC) approach to ensure participation of stakeholders at every level and equity of access to quality services. AMREF used community sensitization sessions, film shows, and stakeholders meetings both at district and sub county level and drama groups to ensure that communities were mobilized for health interventions. It is important to note that Behavioral change communication formed a central pillar to facilitate positive behavior change and capacity building through the provision of correct and relevant information to empower people for more effective decision-making in utilization of health services

The project worked with community based structures (CBOs) and Community Health workers to mobilize communities for utilization of health services through conducting home visits and providing health information. The project supported BCC strategies to disseminate health information through integrated health outreaches, open air discussions, radio talk shows and music and drama shows. Call in Radio talk shows were facilitated by the health workers and the project team and open air discussions were facilitated by both health workers and village health teams. Information Education and Communication (IEC) materials such as posters, fliers and newsletters were used in schools, health facilities and in strategic areas in the communities. A tripartite model was developed involving AMREF, CHWs and CBOs in the management of the project to ensure effective implementation of the project.

**Community open air sessions:** Community health workers and CBOs mobilised communities for the open air sessions. They were organized at parish level. The sessions were aimed at promoting essential attitude change and creation of demand for information and services. The communities’ accessed malaria prevention information in addition to hygiene and sanitation, HIV/AIDS prevention besides testing and counseling. All stakeholders participated in the education meetings. The health workers sensitized communities and shared information on health services sites in the sub counties.

### Partnership with community based structures and CSOs

Partnership is critical for health systems to better meet the needs of the poor. They must place people at the centre; ensure wider use of indigenous knowledge. AMREF has made partnerships with civil society organizations, community based Organisation and the local government team. Communities were empowered to play an active role in the project. The communities’ capacity was built in health education and communication to create an enabling environment at the community level.

**Civil Society Organisations:** AMREF's approach to partnerships was to engage all stakeholders in malaria prevention and control interventions. At national level the project partnered with Malaria Childhood Illnesses Secretariat (MACIS) a civil society organization. Its role was to take a lead in advocacy initiatives of the project at national level. MACIS coordinated all NGOs engaged in malaria and childhood illnesses in the country.

**Community based structures:** The project worked with nine CBOs whose main role was to mobilize for health interventions at community level as well as monitoring the project activities. The CBOs organized themselves into two umbrella associations, which coordinated the different CBOs in the two sub counties. With a limited number of NGOs operating in Nakasongola specifically on malaria the project strengthened the CBOs to promote malaria prevention initiatives in the community. The CBOs work at the grassroots, close to communities, and thus are well placed to offer insight on the ground, and identify community needs. Working with them facilitated community based planning, joint identification and efficient use of resources and allowed for more focused, effective and responsive interventions. The district authorities, health professionals, national NGOs and CSOs operating in the district were involved in the project design, and were involved in the implementation and follow up.

**Community health workers:** AMREF worked with 158 Community health workers (CHWs) in two Sub counties. Each village had two CHWs implementing HBMF (Home Based Management of Fever) strategy. They are voluntary workers who are not paid and chosen by the community to manage diseases at community level. Some of the factors that motivated the CHWs included community recognition and satisfaction derived from seeing children under-five years of age gets healed after treatment.

*Resty Nsamba 30 years old is a community health worker selected by her village, Kyehindula in Nakasongola District in 2008 in Uganda. She has been providing community health services since then. She received various trainings from AMREF, which laid foundation for her impressive record in the village*.

*Resty counsels and refers children and mothers to the health facility in order to save lives and enhance health services. She mobilizes her community for village meetings and sensitizes them on malaria prevention and shares data she has collected in the month*.

*At the same time she is a chairperson of a local AMREF supported CBO “Ffena Kunabegereka”. The CBO is organized by local women and delivers educational messages through drama shows. They are also managing a revolving fund for women to buy treated mosquitoe nets*.

*These volunteer positions are not Resty's first foray into public service. As a volunteer Resty was originally a community medicine distributor as well as an immunization mobiliser for 5 years. When asked why she has volunteered for so many years. She had this to say “When I am needed I go with Great Spirit because I am assisting people in my community; I am ready to do the work.”*

**Behavioral communication and change in malaria prevention:** AMREF adopted the National Malaria Control Program BCC strategy to ensure that the populace accesses the right messages geared towards eliciting positive behavioral practices to prevent malaria. The outcomes of the BCC intervention in this project was to enable beneficiaries increase their knowledge for prevention of malaria, proper utilization of long lasting insecticide treated nets (LLINS) and seeking prompt treatment of malaria/fever within 24 hours to reduce the burden of malaria in project sub-counties. It is important to note that the strategy needs to be complimented by other approaches for effective prevention of malaria such as use of insecticide treated nets, indoor residual spraying and larviciding and environment management. Prevention of malaria is considered an important strategy for reducing the high mortality rates in the country.

**Development of IEC materials:** National Malaria Control Program (NMCP) has developed IEC materials over time. AMREF liaised with the MOH health promotion department and accessed soft copies of IEC materials, which were used to produce posters, fliers, stickers and CHWs training kit. This process was cost effective since some of the steps considered to be important; such as development of key messages, translation of the messages, pre-testing and designing the materials were not done.

*Nabirye Mary lives in Njeru village in Nakitoma Sub County in Uganda. She is a mother 10 months old twins and a 4-year old, all whom frequently suffered from malaria. She is one of the community members who were reached by Community health workers through house to house visits. The CHW used posters and flier's materials to disseminate malaria prevention during the house to house visits, The CHW discovered that she did not own any mosquito net, when the children fell ill, she would use traditional herbs. This was attributed to lack of money to take her children to the nearest health facility for treatment. Mary used her new found knowledge to protect her children from diarrhea and malaria. She bought herself one mosqutoe net and benefited from the nets distributed to children under five and pregnant by AMREF. She now owns 4 treated nets and her children no longer fall ill all the time as it was the case. When AMREF staff visited her home she could not hide her joy*.

## Results

This section summarizes the key outcomes, contributing to impacts and lessons during the implementation of the Nakasongola malaria project. The end line used a Cross-sectional survey design and one sub-county (Wabinyonyi) was used as a control area in order to derive the true impact of the project activities. Comparison between the baseline and end line demonstrated outcomes of the interventions.

**Figure 1:**
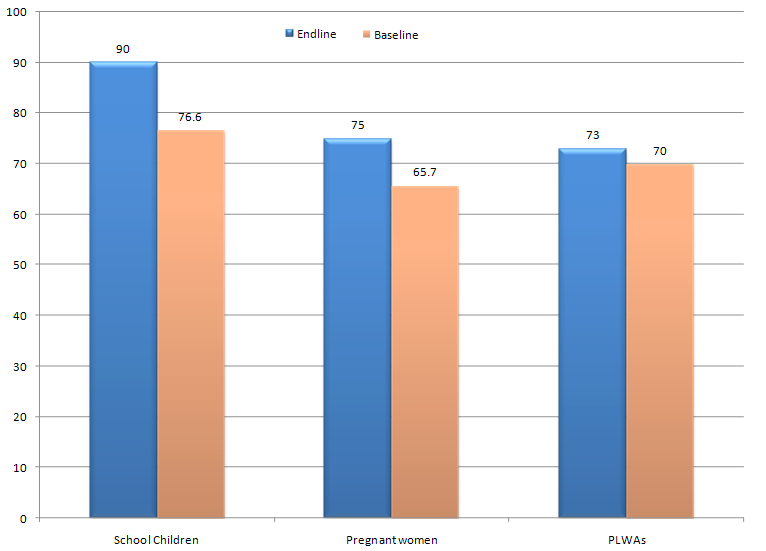
Graph showing malaria knowledge levels among school going children, pregnant women and PLWAs in project operation area (Source: AMREF, Malaria Prevention and Control Project Report)

Community Based Organizations and CHWs spearheaded the behavioral communication and change interventions in the community. There was an increase in knowledge among school going children ages (10-24) from 76.6% at baseline to 90%, the pregnant women from 65.7% to 73%, and from 70% to 75% among PLWAs. Figure 1 illustrates variations in the level of knowledge among different target groups of the project. Overall, the knowledge level was high among schoolchildren compared to pregnant women and PLWAs.

**Figure 2:**
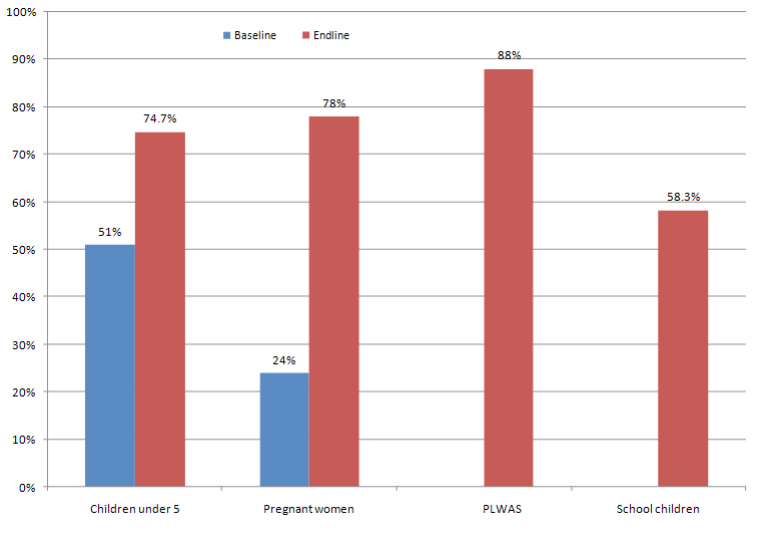
Use of insecticide treated nets (ITNs) among target groups in Kakooge and Nakitoma Sub Counties (Source: AMREF, Malaria Prevention and Control Project)

Community health workers were at the centre of disseminating information on the use of mosquitoe nets through house to house visits, community open air sessions. There was increased utilization of long lasting insecticide nets from 51% to 74.7% among children under- five and from 24% to 78% among pregnant women at the end of the project. There was no baseline to compare with; however ITN utilization rate among school children was 58.3% and 88% among PLWAs respectively. These two utilization rates of nets (Figure 2) were higher than the national average of net utilization (42%) [16]. It is important to note that net utilization is still a challenge despite an increase in certain target groups. The communities are still poor and they still rely on hand outs from development partners.

Health seeking behavior among women increased from 75% to 80% at health facility level. There was a decline in male involvement in antenatal clinic (ANC) related activities at health facilities from 88% at the start of to 60.1% at the end of the project. The project team observed that BCC messages concentrated on female/mother's roles and less on male involvement. It was not possible to establish prompt treatment within 24 hours because the health facility data was not indicative of the onset of fever and the VHTs in the community were only referring patients but not treating due to lack of drugs.

The Malaria Project and MACIS helped to address the challenges of weak coordination, collaboration and advocacy among NGOs/CBOs involved in malaria and other healthcare interventions. As a result, two umbrella organisations (Nakitoma Umbrella Community Development initiative and strengthening Rural Community Development Initiative in Kakooge) were formed to unite the various CBOs operating in Kakooge and Nakitoma Sub counties. The two umbrella associations were responsible for the coordination of CBOs within their area, share reports with AMREF, MACIS, district community development and health departments. Subsequently by working as association they were charged with minimization of replicating interventions and strengthening advocacy initiatives in the communities. The umbrella associations were charged with resource mobilisation within the district and at national level. The associations in Kakooge Sub County secured funding from Program Accessible health Communication and Education (PACE) a national CSO in Uganda as a result of CBO's unification.

Health system strengthening was enhanced through building capacities of more than 25 health workers in the use of rapid diagnostic tests and management of malaria at health facility level for better delivery of health services

## Discussion

### Lessons learnt

Partnership with CBOs enhances their stature in communities and enables them manage their health and stimulate positive health behavior change in the community. Community health workers are significant in management of community health interventions once facilitated and monitored, they cause positive change in health seeking behaviour by dissiminating health messages and conducting household monitoring. Integration of behavioral communication and change interventions (health education campaigns radio talks, films and drama shows) and provision of health services improves their uptake among children, pregnant women and People Living with Aids. BCC materials should also include information on male participation in health intervention this would contribute to scaling up of ANC attendance and utilization of health services.

### Recommendations

Behavioral change communication strategy should complement other malaria prevention strategies for effective results. The development of BCC/IEC materials (posters, fliers, newsletters) should be well planned in order for the communities to benefit from the IEC materials. Community health workers are essential in the implementation of health interventions; there is need to facilitate them adequately through frequent monitoring, provision of work kits and drugs and bicycles to enable them carry out their work diligently. This is the only approach to sustain community interventions.

More effort is needed scale up intermittent presumptive treatment uptake among pregnant women. CHWs should be equipped with Fansidar (SP) so that IPTp is managed at household level. Government should ensure the supply of adequate essential drugs and equipment in health centers as a way of motivating communities to seek treatment within 24 hours of the onset of fever.

## Conclusion

Malaria control strategies in Uganda and other regions of the world can learn from AMREF's experience. The key message is that Behavior Change Communication approach is likely to be more effective if carried out in an integrated fashion with other malaria control strategies; and in partnership with existing community structures.
